# Current trends in macromolecular model refinement and validation

**DOI:** 10.1107/S2059798323010823

**Published:** 2024-01-01

**Authors:** Melanie Vollmar, Robert Nicholls, Svetlana Antonyuk

**Affiliations:** a European Bioinformatics Institute (EMBL-EBI), Main Building, A2-34, Wellcome Genome Campus, Hinxton CB10 1SD, United Kingdom; b MRC Laboratory of Molecular Biology, Francis Crick Avenue, Cambridge CB2 0QH, United Kingdom; c Institute of Integrative Biology, University of Liverpool, Crown Street, Liverpool L69 7ZB, United Kingdom

**Keywords:** CCP4 Study Weekend 2022, model refinement, model validation

## Abstract

For structural biology practitioners a number of techniques, such as X-ray/neutron/ electron crystallography and cryo electron microscopy (cryo-EM), are available to determine the 3D atomic structure of a macromolecular structure. Any one of these techniques can be applied to produce an initial model, comprising atomic positions and their associated uncertainties, which is fitted to the experimental data, such as a set of diffraction intensities or an electrostatic potential map. By optimizing the agreement between model and data, refining the atomic coordinates and other model parameters, a researcher gains unique and novel insights into a macromolecule’s architecture, often facilitating inferences and further hypotheses regarding function and mechanism. Crucially, this optimization process is limited by the quality of the acquired data and, to a lesser extent, by the algorithms and their implementations in data analysis software. Depending on the scientific question asked by a researcher, the necessary data should have been acquired by the best suited technique, and refinement and validation procedures selected to produce the highest possible data analysis results and the best quality structural model, given the purpose of the experimental analysis.

The CCP4 Study Weekend 2022 looked at the currently available refinement and related techniques that can be used to facilitate optimization of the agreement between model and data. We considered applications that help a researcher to achieve the best possible data analysis results for addressing their scientific question, and how to avoid false or over interpretation through model validation.

The first day started with a session on practical considerations, with Eleanor Dodson giving a general practical overview of refinement procedures and various considerations that should be taken into account. Airlie McCoy (twinning and other crystal pathologies) and Elspeth Garman (radiation damage) then presented an introduction to common issues that can be encountered when refining a protein structure determined using X-ray diffraction. Different pathologies require additional treatment and careful interpretation of the model-to-data fit in order to produce a high quality structural model. Marcus Fisher discussed the effects of room temperature (versus cryo-) crystallography on structural models, and benefits in the context of structure-based ligand discovery. The associated contribution describes the recently developed tool *Flexr*, which helps with modelling and interpretation of flexible areas that are characterized by weakly defined density. Modelling such flexibility allows for the study of a protein’s dynamics and may even contribute to understanding a mechanism of action. The session was closed by Andrea Thorn who presented common modelling errors identified for structures in the PDB. She explained that such errors are far from being an exception and that structural biologists should aim to not repeat the same errors and avoid error propagation.

The second session was centred around additional information to be used in the refinement process, in particular restraints, which are necessary to describe the correct geometry of chemical compounds. After Oleg Kovalevskiy explained how limits in resolution affect refinement, and what techniques are available to facilitate stabilizing refinement at lower resolutions, Bohdan Schneider detailed how nucleic acids should be described so they can be properly modelled in, for example, nucleic acid–protein complexes. The last part of this session introduced three software tools that can be used to create restraint dictionaries in order to appropriately describe the stereochemical geometry of molecular components, as required for successful macromolecular model refinement. Andrey Lebedev presented the tool *AceDRG* in *CCP4*, Clemens Vonrhein gave an introduction to *Grade* in *GlobalPhasing* and Nigel Moriarty explained the usage of *eLBOW* in *Phenix*.

On the second day of the conference, the first session focused on new developments and approaches to refinement. The first speaker, Helen Ginn, presented the software *Vagabond* and how it was used to refine and analyse large numbers of models relevant to the SARS-CoV-2 pandemic. Kevin Cowtan then detailed how fast and efficient early stage refinement can be performed using the shift field refinement method. The session was closed by a presentation from Lucrezia Catapano, who described an extension to the package *REFMAC5* that enables refinement against neutron diffraction data, accounting for both nucleus and electron positions for hydrogen atoms.

The second session of day two focused on four commonly used modern X-ray crystallographic macromolecular refinement programs. Keitaro Yamashita gave an overview of the *REFMAC5* package and how it can be used to combine data from a number of sources during refinement. This was complemented by describing the new tool *Refmacat* for crystallographic model refinement. *Refmacat* is part of *Servalcat*, which coordinates refinement by *REFMAC5* using the toolbox *Gemmi*. Gerard Bricogne presented the essential refinement features of *Buster* from *GlobalPhasing*, followed by Pavel Afonine who described the different tools and options available in *phenix.refine* of the *Phenix* suite. The session was closed by Isabel Usón providing an overview of the options and tools in the package *SHELXL*.

The final session of day two looked at ways to integrate data from various experimental sources in model refinement. Dorothee Liebschner described how one can combine data from X-ray, neutron and electron diffraction when refining a model, as implemented in the *Phenix* suite. As all three methods use different approaches to determine the atomic positions within a protein structure, they provide complementary information when building a model. Lukas Gajdos then gave further details on how to make the best use of information derived from neutron diffraction data. Following was Dennis Della Corte, who gave an introduction in how molecular dynamics simulations can be leveraged to minimize the energy landscape of atomic models. In particular, he described how this can be achieved using limited computational resources and a team of inexperienced researchers, provided such an effort is coordinated with good project planning and clear guidelines. The final talk in this session was by Elke de Zitter, who presented the tool *Xtrapol8*, which was developed in the field of time-resolved crystallography. The tool allows for the refinement of low-occupancy states of proteins and small molecules, as may be found transiently in a reaction mechanism step.

On the third and final day of the meeting, the first session was looking at interactive model building by a researcher as well as automated pipelines that will aim to produce an initial model to be subsequently further optimized. Paul Emsley gave an introduction to the model building package *Coot* and described some of the most essential tools within. Two automated model building pipelines followed. Firstly, Victor Lamzin presented the package *ARP/wARP* and how it can be used to automatically build models at a range of resolutions, not just using X-ray diffraction data but also using electrostatic potential maps derived from cryo-EM. The second package, *Buccaneer*, was presented by Paul Bond. In particular he focused on the use of machine learning in the *ModelCraft* pipeline to inform backbone and side chain pruning during each building cycle. Finally, Tristan Croll showed how the program *ISOLDE* can be combined with *phenix.process_predicted_model* to work with predicted protein structure models, such as those produced by *Alpha-Fold2*. The created pipeline can be used to generate fragments to be docked into experimental data for subsequent refinement.

The final two sessions of the meeting focused on structure validation and error remediation. Robbie Joosten gave an overview of the resource *PDB-REDO*, which applies a number of processing and standardization tools to structures deposited to the PDB and aims to create a new collection of curated and improved models that can, for example, be used to guide model building and refinement. Jane Richardson gave a summary of the collection of checks that are implemented in the validation tool *MolProbity*, and discussed how the produced statistics can be used to guide improvement of models during the refinement procedure, most importantly identifying problematic regions that require special attention prior to deposition to the PDB. The validation tool *Privateer* was presented by Jon Agirre, who demonstrated how glycan chemistry should be modelled in protein structures. Glycans are not only essential binding partners in an active site or are part of a reaction mechanism, but far more frequently and importantly represent a large number of post-translational modifications. These are essential in cell–cell recognition and signalling and, crucially, have to be modelled correctly. Gerard Kleywegt then provided an opinion piece on why new technologies such as AI and structure prediction do not obsolete structural biologists. He argued the opposite, that structural biologists are essential to ensure that false information and incorrect assumptions are not drawn from automatically generated results, and that critical human thinking is used to thoroughly scrutinize findings. The second half of the session moved towards structure deposition, with David Armstrong describing the current processes at the PDB. He also discussed the impact of the PDB not just as a data repository but as a data resource, in light of the new challenges for structural biology created by structure prediction tools such as *Alpha-Fold2*. Thereafter, Grzegorz Chojnowski presented two tools that use machine learning to aid protein sequence assignment and validation: *FindMySequence*, which identifies and validates sequence assignments in X-ray crystallography and cryo-EM, and *CheckMySequence*, which validates sequence assignment in cryo-EM models by searching for register errors. Sequence shifts and erroneous modelling are relatively common yet were previously hard-to-detect problems, in particular at lower resolutions. Filomeno Sanchez-Rodriguez described the usage of evolutionary co-variance in model validation as implemented in the tool *ConKit*. This tool involves predicting inter-residue distances using a deep learning algorithm exploiting information about evolutionary co-variance. The final contribution to the last session was by Eugene Krissinel who described how *CCP4* can be run in various deployment scenarios, including within a cloud computing environment, and how the web-based *CCP4 Cloud* resource allows for eased workflows and streamlined processes.

Two additional publications have been contributed to the special issue arising from the What’s New In CCP4 session, which was held on the morning of day two and focused on the use of predicted models in computational structural biology. Adam Simpkin *et al*. reported on the various ways that predicted models can be used within the *CCP4* suite in general. This not just covered model building and refinement, but also included phasing. Ana Medina *et al*. further described how the tool *ARCHIMBOLDO-SHREDDER* can make use of predicted models in phasing.

As the field of structural biology has seen some fundamental changes due to new developments, those in structure prediction in particular, a number of thought-provoking keynote lectures were presented, challenging established views and ideas. Kevin Cowtan opened the conference with their challenge to the stereotypical view on what a programmer or software developer looks like and contemplated the history of perceived gender roles within the field. Dale Tronrud gave a summary on the golden era of structural biology in the 1970s and what can be drawn from the experiences there to prepare for future changes. Bernhard Rupp discussed why model validation is important, what the current state of validation is like and how new developments such as predicted models with near perfect geometry challenge established validation processes and enforce the development of new methods. The final keynote lecture was presented by John Helliwell who pointed out that the raw (diffraction) data from an experiment represents the ultimate ground truth against which all analysis and refinement should be carried out. As such it is vital to preserve such raw data in case questions about result interpretation need to be answered in detail, and indeed to ensure that workflows are properly recorded adhering to FACT and FAIR principles.

Finally, an up-to-date overview of the *CCP4* suite by Jon Agirre *et al*. was included in this special issue. The complete special issue can be found at https://journals.iucr.org/special_issues/2023/CCP42022/.

Each data processing step increases uncertainty – errors propagate throughout the structure determination process. Rationalizing which model features should be considered reliable, and which are merely the result of random or systematic error due to data collection and analysis, remains a serious challenge during the model building and refinement process. Various quantitative measures are used to assess data/ model quality throughout the procedure, yet assessment and avoidance of error propagation remains difficult. Global refinement and model statistics do not directly relate to local model correctness, which is essential for drawing accurate biological conclusions through model analysis. Continued advancements in model building, refinement and validation software aim to ease the final stages of the structure determination process, resulting in as reliable and accurate models as possible.

